# Translational Proteomics Analysis of Anthracycline-Induced Cardiotoxicity From Cardiac Microtissues to Human Heart Biopsies

**DOI:** 10.3389/fgene.2021.695625

**Published:** 2021-06-15

**Authors:** Nhan Nguyen, Terezinha Souza, Marcha C. T. Verheijen, Hans Gmuender, Nathalie Selevsek, Ralph Schlapbach, Jos Kleinjans, Danyel Jennen

**Affiliations:** ^1^Department of Toxicogenomics, GROW School for Oncology and Developmental Biology, Maastricht University, Maastricht, Netherlands; ^2^Genedata AG, Basel, Switzerland; ^3^Swiss Integrative Center for Human Health, Fribourg, Switzerland; ^4^Functional Genomics Center, ETH Zurich and University of Zurich, Zurich, Switzerland

**Keywords:** WGCNA, cardiotoxicity, anthracyclines, proteomics, drug side-effect

## Abstract

Anthracyclines, including doxorubicin, idarubicin, and epirubicin, are common antitumor drugs as well as well-known cardiotoxic agents. This study analyzed the proteomics alteration in cardiac tissues caused by these 3 anthracyclines analogs. The *in vitro* human cardiac microtissues were exposed to drugs in 2 weeks; the proteomic data were measured at 7 time points. The heart biopsy data were collected from heart failure patients, in which some patients underwent anthracycline treatment. The anthracyclines-affected proteins were separately identified in the *in vitro* and *in vivo* dataset using the WGCNA method. These proteins engage in different cellular pathways including translation, metabolism, mitochondrial function, muscle contraction, and signaling pathways. From proteins detected in 2 datasets, a protein-protein network was established with 4 hub proteins, and 7 weighted proteins from both cardiac microtissue and human biopsies data. These 11 proteins, which involve in mitochondrial functions and the NF-κB signaling pathway, could provide insights into the anthracycline toxic mechanism. Some of them, such as HSPA5, BAG3, and SH3BGRL, are cardiac therapy targets or cardiotoxicity biomarkers. Other proteins, such as ATP5F1B and EEF1D, showed similar responses in both the *in vitro* and *in vivo* data. This suggests that the *in vitro* outcomes could link to clinical phenomena in proteomic analysis.

## Introduction

Anthracycline (ANT) is a group of well-known chemotherapeutic agents consisting of thousands of analogs; the commonly used analogs are doxorubicin (DOX), epirubicin (EPI), and idarubicin (IDA). DOX belongs to the first generation of ANT, while EPI and IDA represent the second generation of ANTs (Muggia and Green, 1991; Minotti et al., 2004). DOX is an essential drug in the treatment of multiple cancer types such as acute lymphoblastic leukemia, nephroblastoma, diffuse large B-cell lymphoma, and Hodgkin lymphoma, in both children and adults (WHO, 2019a,b). EPI, as a derivative of DOX, has a similar spectrum of activity compared to DOX. IDA, a derivative of daunorubicin, has shown more potency in antitumor activity, especially, its efficacy in multidrug resistance compared to other ANTs (Kuffel et al., 1992; Minotti et al., 2004).

Despite being widely used in cancer treatments, ANTs have been defined as cardiotoxic agents. Multiple cohort studies have shown that ANTs exposure dose-dependently increases the risk of cardiac disorder in cancer survivors (Mulrooney et al., 2009; Bates et al., 2019). Empirical studies have confirmed that cardiac muscle function is impaired by ANTs. While ANTs increased the maximal tension of the cardiac fibers in rat hearts, other chemotherapeutic drugs such as taxol and fluorouracil did not (Bottone et al., 1997). The cardiac toxicity limits the application of ANTs in clinical treatment. The first generation of ANTs, such as DOX, has been considered more cardiotoxic than the later generation, including EPI and IDA (Minotti et al., 2004). For example, in rat hearts, the degree of the maximal tension elevation by the same concentration (20 μM) of DOX was higher than that of EPI and IDA (Bottone et al., 1997). In order to reduce the cardiac disorder risks, the maximum cumulative doses of DOX and EPI used are recommended to be 450–600 mg/m^2^ or 900 mg/m^2^, respectively (Ryberg et al., 1998; Minotti et al., 2004). While the maximum cumulative dose of IDA for cardiac safety has not been defined, patients treated with IDA cumulative doses in a range of 150–400 mg/m^2^ showed a low probability of cardiotoxicity (Electronic Medicines Compendium (EMC), 2020). In a retrospective study on acute myeloid leukemia and myelodysplasia patients, the probability of IDA-related cardiomyopathy was 5% at a cumulative IDA dose of 150–290 mg/m^2^ (Anderlini et al., 1995).

Although a range of studies has investigated how ANTs induce heart toxicity, the distinct molecular mechanism leading to their adverse cardiac effects remains unclear. The conventional paradigm was that reactive oxygen species generated by ANTs cause damage to multiple cellular components. However, combinations of ANTs with multiple antioxidants have failed to promote cardioprotection (Šimůnek et al., 2009), so ANTs’ toxic mechanism has been considered as complex and multifactorial (Geisberg and Sawyer, 2010). Emerging perspectives have suggested that ANTs can modulate growth factor receptors (ErbB2 and ErbB4), β2 adrenergic receptor (β2AR), and Toll-like receptors (TLR2 and TLR4). Therefore, ANTs may alter particular signaling pathways including PI3K, NF-κB, and GATA4 pathways, and then impact cardiac immune functions, cardiac contractility, and cardiomyocyte survival (Ghigo et al., 2016). Even though recent studies have updated explanations for ANT-induced cardiotoxicity, full insight into the ANT mode of action is still missing.

Some studies have focused on protein activities prompted by ANT exposure because the change of the proteome, a key factor of cell functions, would directly influence cellular function and survival. In addition, the protein biomarkers can transposable to clinic application. A study in isolated adult rat cardiomyocytes showed that the inhibition of membrane-bound calcium-independent phospholipase A2 (iPLA2) by ANTs restricts the recuperative capacity of cardiomyocytes (Swift et al., 2003). Another study, also performed in rats, demonstrated that the upregulation of SIRT1, a member of the Sirtuin protein family, improves cardiac function after DOX treatment (Cappetta et al., 2016). A further study has explored the changes in protein levels in association with mRNA levels in the mitochondria of rat hearts after DOX treatment. It suggests that the alterations from transcript to protein level serve as early acute markers of cardiac-specific mitochondrial toxicity (Pereira et al., 2019). Thus, the alteration of protein profiles could reveal different aspects of ANTs-induced heart failure and thereby provide a better understanding of ANTs mechanism of cardiotoxicity. Overall, researchers so far mainly focused on the proteome upon DOX treatment, while little attention has been paid to the protein expression induced by other ANT analogs such as EPI and IDA.

In this study, we investigated the proteome-wide profiles under ANTs exposures in an *in vitro* cardiac microtissue experiment and heart failure biopsies from ANTs treated patients. ANTs, as a drug family, can share a common mechanism of toxicity and reshape cardiac protein expressions. Furthermore, DOX, EPI, and IDA can have their variant adverse effects on cardiac tissue, which could also be captured by the protein expression alterations. The ANT-affected protein groups were acquired in the *in vitro* data and human cardiac biopsies data. The association of these proteins with heart failure, as well as their protein functions and protein interactions were appraised. By combining the 2 datasets, we aimed to translate the protein alternation patterns from the *in vitro* experiments to the protein expression in heart failure patients. Hence, this study accommodates a deeper understanding of ANTs-induced cardiotoxicity on the proteomic level and suggests some new proteins as prominent targets for ANTs-related heart failure study.

## Materials and Methods

This study used the proteomics data from the Hepatic and Cardiac Toxicity Systems modeling project funded by the European Union Seventh Framework Programme (FP7/2007-2013).

### Cardiac Microtissue Culture and Treatment

**FIGURE 1:**
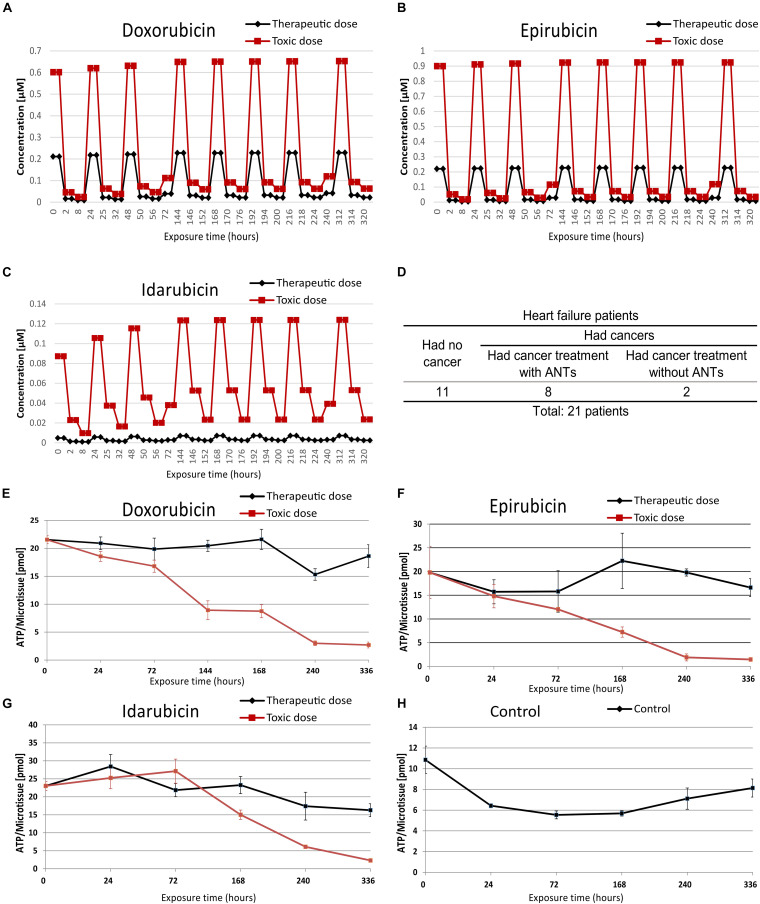
The *in vitro* microtissue and biopsies samples information. The therapeutic and toxic concentration profiles of doxorubicin **(A)**, epirubicin **(B)**, and idarubicin **(C)** that were used in the cardiac microtissue experiment. Profiles of biopsy samples were taken from heart failure patients **(D)**. The ATP contents of microtissues were treated with doxorubicin **(E)**, epirubicin **(F)**, idarubicin **(G)**, and control sample **(H)**.

The human cardiac microtissues (3D InSight^TM^ Human Cardiac Microtissues from InSphero) were used as an *in vitro* model. It contained 4,000 iPS-derived human cardiomyocytes from a female Caucasian donor and 1,000 cardiac fibroblasts from a male Caucasian donor. The microtissues were cultured in 3D InSight^TM^ Human Cardiac Microtissue Maintenance Medium (InSphero) and exposed to DOX, EPI, and IDA for 2 weeks. Stock solutions of the drugs were dissolved in 0.1% DMSO, then were rectified to their interstitial heart concentrations over time that calculated by reverse physiologically based pharmacokinetic (PBPK) modeling according to dose administration (Kuepfer et al., 2018). Per drug, the microtissues were exposed to 2-based repetitive dosing profiles (Figure 1 and Supplementary Table 1), namely a therapeutic dose and a toxic dose: the therapeutic dose reflected the clinical dose and the toxic dose was the IC20 value based on the ATP production (cell viability) previously determined after 7 days of exposure (Verheijen et al., 2018). Every weekday, the sample medium was renewed 3 times corresponding to the drug concentration profile at 2, 8, and 24 h calculated by the PBPK module (Supplementary Table 1). The microtissues were harvested after 2, 8, 24, 72, 168, 240, and 336 h of ANTs exposure with 3 replicates per dose, except for samples treated with the toxic dose of IDA. The IDA-treated samples were harvested until 168 h of exposure because of substantial cell death at later time points. The IDA-treated samples after 2 and 24 h of exposure had only 2 replicates. In total, the *in vitro* testing generated 139 samples.

### Sample Preparation of Human Cardiac Microtissues

After collection, cardiac microtissues were resuspended in 100 μl lysis buffer containing 8 M Urea, 1 mM Dithiothreitol, 0.1 M Ammonium bicarbonate, pH 7.8. After four freeze-thaw cycles, the samples were centrifuged at 16,000 × *g* for 15 min at 4°C and protein concentrations were assessed with the Qubit^TM^ Protein Assay Kit (Invitrogen, Molecular Probes). Protein isolates were then submitted to in-solution digestion (Selevsek et al., 2011) or Filter Aided Sample Preparation (FASP) (Wiśniewski et al., 2009). Protein digestions were stopped by adding formic acid to a final concentration of 1%. The peptides were cleaned up using Sep-Pak tC18 cartridges (Waters) according to the manufacturer’s instructions and eluted with 60% ACN and 0.1% formic acid (Sigma-Aldrich, United States).

### ATP Measurement

After the microtissue collection, ATP content in microtissues was prepared as described in Verheijen et al. (2019). In short, the ATP level of the microtissues (*n* = 4) was measured using Promega’s CellTier Glo 3D (Cat #G9683) according to the manufacturer’s instructions. The luminescence was measured after 30 min incubation with the luciferase reagent (Verheijen et al., 2019).

### Human Cardiac Biopsy Samples

Cardiac biopsies were taken from 21 heart failure patients including 11 patients who had no cancer history as control subjects, 8 patients who had cancer and received medications including ANTs, and 2 patients who had cancer and received medications without ANTs (Figure 1 and Supplementary Table 2). The Medical Ethics Committee of Maastricht University Medical Center approved the patient biopsies collection. Five biopsies (2 ANT-treated patients and 3 control patients) were used in a pilot study to establish the sample preparation workflow including the sample collection, sample processing, and data measurements. The sample preparation workflow was then applied to the remaining biopsies.

### Sample Preparation of Human Cardiac Biopsies

Samples were lysed and digested using a Barocycler NEP2320 (Pressure BioSciences) at 33°C (Guo et al., 2015). Briefly, each sample 1 (±0.1) mg was lysed with a buffer containing 8 M Urea, 0.1 M Ammonium bicarbonate, and complete protease inhibitor (Roche), in combination with a cycling program of 50 s of ultrahigh-pressure (45,000 p.s.i.) and 10 s of ambient pressure (total of 60 pressure cycles). Protein reduction and alkylation were performed with 10 mM tris (2-carboxyethyl)phosphine and 40 mM iodoacetamide, respectively. Protein digestions were performed sequentially with Lys-C and trypsin using PCT with a cycling scheme of 50 s at 20,000 p.s.i. and 10 s at ambient pressure. Lys-C digestion was carried out in 6 M urea for 45 cycles, whereas trypsin digestion was performed in 1.6 M urea for 90 cycles. Protein digestions were stopped by adding trifluoroacetic acid (TFA) to a final concentration of 1%. The peptides were cleaned up using reverse-phase cartridges Finisterre SPE C18 (Wicom International AG) according to the manufacturer’s instructions.

### Mass Spectrometry (MS) Measurements

Digested peptides from cardiac microtissues and cardiac biopsies were submitted to an Orbitrap Fusion mass spectrometer (Thermo Fisher Scientific) coupled to a NanoLC-2D HPLC system (Eksigent, Dublin, CA) or EASY-nLC 1000 system (Thermo Fisher Scientific, Germany). Samples were loaded onto a self-made column (75 μm × 150 mm) packed with reverse-phase C18 material (ReproSil-Pur 120 C18-AQ, 1.9 μm, Dr. Maisch HPLC GmbH) when coupled with the EASY-nLC 1000 system and onto an Easy-Spray Column (75 μm × 500 mm) packed with reverse-phase C18 material (Silica 100 Å, 2 μm) when coupled with the NanoLC-2D HPLC system. Peptides separated with a linear gradient of acetonitrile/water, containing 0.1% formic acid, at a flow rate of 300 nl/min. A gradient from 5 to 30% acetonitrile in 60 min was used. The mass spectrometer was set to acquire full-scan MS spectra (300–1,500 m/z) at 120,000 resolution at 200 m/z; the precursor automated gain control (AGC) target was set to 400,000. A charge-state screening was enabled, and precursors with +2 to +7 charge states and intensities >5,000 were selected for tandem mass spectrometry (MS/MS). Ions were isolated by using the quadrupole mass filter with a 1.6 m/z isolation window. Wide quadrupole isolation was used, and the injection time was set to 50 ms. The AGC values for MS/MS analysis were set to 5,000 and the maximum injection time was 300 ms. HCD fragmentations were performed at normalized collision energy (NCE) of 30%. MS/MS was detected in the ion trap in centroid mode. Precursor masses previously selected for MS/MS measurement were excluded from further selection for 25 s, and the exclusion window was set at 10 ppm.

### Peptide/Protein Quantification and Preprocessing Data

Raw MS data were processed using Genedata Expressionist^®^ software v.11.0. In short, after noise reduction and normalization, LC-MS peaks were detected and their properties calculated (m/z and RT boundaries, m/z and RT center values, intensity). Individual peaks were grouped into clusters and MS/MS data associated with these clusters were annotated with MS/MS Ions Search (Mascot 2.6) using Peptide Tolerance: 10.0 ppm, MS/MS Tolerance: 0.50 Da, Max Missed Cleavages: 2, and database: Uniprot Swiss-Prot 29062016, Taxonomy Homo sapiens (human). Results are validated by applying a threshold of 5% normalized False Discovery Rate (FDR). Protein interference was done based on peptide and protein annotations. Redundant proteins were ignored according to the Occam’s razor principle, and at least 2 peptides were required for positive protein identification (shared peptides were ignored). Protein intensities were computed using the Hi3 method. A maximum of the top 3 peptides per protein (based on the average intensity across samples) was used in the calculation. If a peptide was identified in multiple charges (2+, 3+, and 4+) and modification states [Carbamidomethyl (C), Deamidated (NQ) or Oxidation (M)], values were consolidated into a single peptide intensity. The volume of a peak was computed as the area under the intensity curve inside the peak region. The area under the intensity curve is subdivided into trapezoids at the data points according to the trapezoidal rule. Volume is robust to different scan rates and takes more information (data points) into account. The intensities were log2 transformed.

To normalize the *in vitro* proteomic data, the log2 transformed values of the control samples were shifted to the median of the medians determined by a reference group consisting of the proteins found in all these control samples. For every ANT treatment and for each time-point, the common protein set between the controls and the ANT-treated samples was determined. The median of the medians of the (in general 3) normalized control samples was determined using this common protein set between the controls and the treatment samples. The data from the samples of the ANT treatments were shifted to these medians.

To normalize the heart biopsies data, the log2 transformed values of each sample were shifted to a common median which was determined using only the proteins present in all samples.

### Proteomic Data Analysis

**FIGURE 2:**
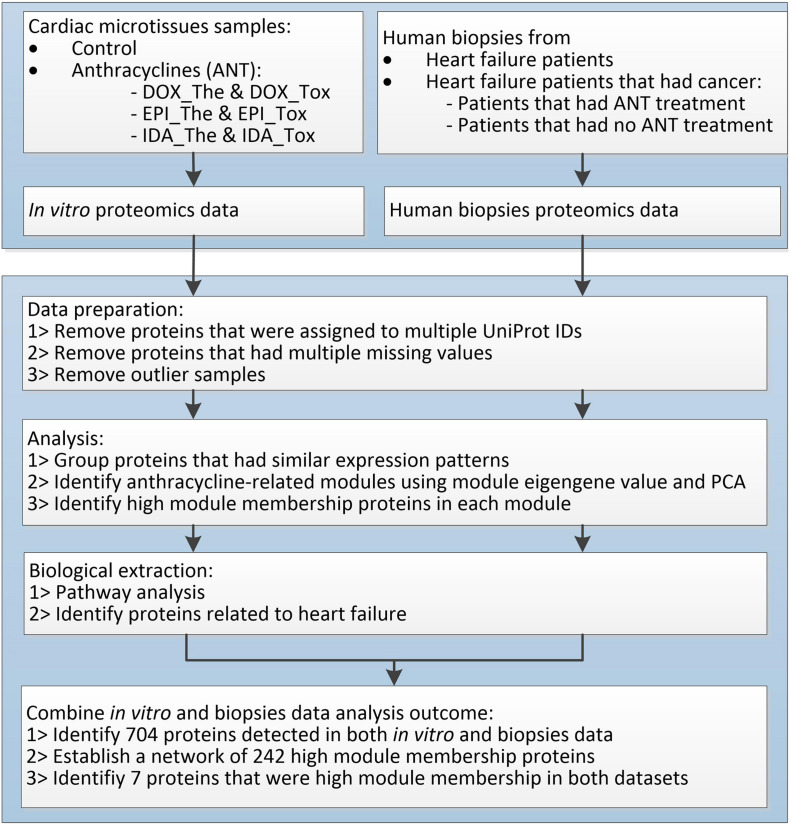
The proteomic data analysis workflow.

The log2 transformed normalized values were standardized to 6 decimal digits and analyzed separately between *in vitro* dataset and biopsies dataset using the same workflow (Figure 2). In each dataset, proteins that matched multiple UniProt IDs were removed. The protein expression patterns were analyzed using the weighted correlation network analysis (WGCNA) package for finding modules of highly correlated proteins (Langfelder and Horvath, 2008) in R (version 3.5.3, released on 11th March 2019). Proteins that had over 50% missing values across samples were removed using the default filter of the goodSamplesGenes function in WGCNA. Hierarchical clustering sample trees were built from the remaining proteins. Thereafter, clustering protein trees were established based on the similarity of protein expression profiles across samples using the adjacency function with a signed network. In the *in vitro* data, power = 2 was used to provide a protein clustering tree that had topological overlap measures *R*^2^ = 0.64 and mean connectivity = 59.6. In the human biopsies data, power = 5 was used to provide a protein clustering tree with similar features as the *in vitro* data tree (topological overlap measures *R*^2^ = 0.73, mean connectivity = 56.9). Proteins in the branches of protein clustering trees were divided into modules (groups) named by colors using the cutreeDynamic function (distM = dissTOM, deepSplit = 3, pamRespectsDendro = FALSE, minClusterSize = 30). These modules were merged when the difference between their module eigengene profiles was less than 0.25. In particular modules, proteins with high module membership (≥0.8), which demonstrate the strong impact of these proteins in the module, were considered as weighted proteins.

Module eigengene values (the first principal component) and the principal component analysis (PCA), which can reveal the protein expression variation between samples, were used to defined ANT-affected modules. The biological pathways of proteins in ANT-affected modules were explored by performing over-represented pathways analysis using ConsensusPathDB with *p*-value and *q*-value < 0.01 (Kamburov et al., 2011). Simultaneously, a reference of all proteins associated with a query term “heart failure” (572 proteins) in the DisGeNET database, a collection of genes and variants associated with human diseases, version 7.0, released on 4th May 2020 (Piñero et al., 2019), was used to recognize the association of detected proteins to heart failure.

Proteins presented in both *in vitro* dataset and biopsies dataset were defined. The UniProt IDs of proteins, which were weighted proteins in at least one dataset, were used to build a protein network using the BisoGenet app with Homo sapiens species data, with non-adding connection, and other parameters following the default settings (Martin et al., 2010) in the Cytoscape version 3.7.1 (Shannon et al., 2003). The protein-protein interaction information is based on multiple sources comprised of the DIP, BIND, HPRD, MINT, Intact, and BioGrid databases (Martin et al., 2010). Proteins, which were weighted proteins in both datasets, as well as the hub proteins, which have ≥30 connections in the protein network, were identified.

## Results

### Sample Information

The human cardiac microtissues were incubated with either a clinically therapeutic dose or a toxic dose (IC20) of ANT analogs (DOX, EPI, and IDA) using PBPK-based dose profiles (Kuepfer et al., 2018; Figures 1A–C and Supplementary Table 1). When the cardiac microtissues were exposed to ANT conditions in 2 weeks, the proteome was extracted and measured at 7 time points, from 2 to 336 h, except for IDA toxic-treated samples at 240 and 336 h because of substantial cell death. The cardiac biopsies were collected from heart failure patients who had no cancer and heart failure patients who had cancer and previously received ANT treatments or non-ANT treatments (Figure 1D and Supplementary Table 2). The function of mitochondrial was measured via the ATP levels in the *in vitro* microtissues under ANTs exposure (Figures 1E–H). In the samples treated by the same ANT analog, the toxic dose-treated samples had lower ATP levels than the therapeutic dose-treated samples, except for the IDA-treated samples after 72 h of exposure. The ATP measurement emphasized that ANT-induced mitochondrial dysfunction was dose-dependent, especially after long-time exposure.

Proteins in microtissues and human biopsies were detected using LC-MS. A workflow was applied to investigate the effects of ANTs in the heart proteome (Figure 2).

### *In vitro* Data

**FIGURE 3:**
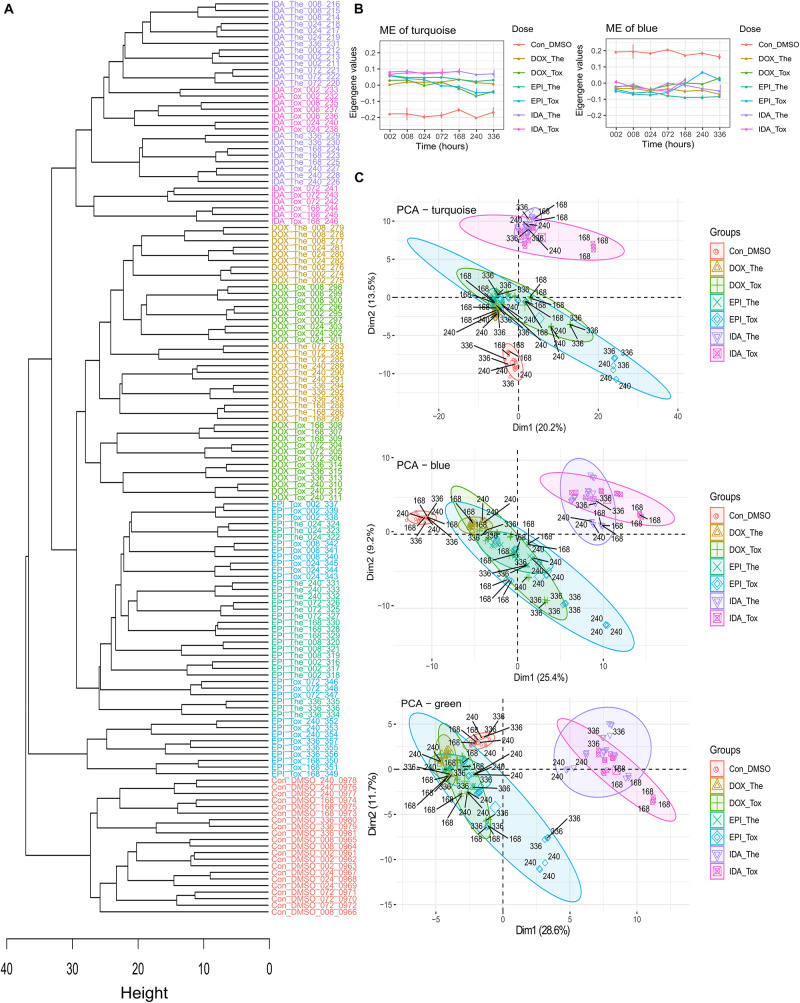
The *in vitro* cardiac microtissue proteomics data analysis. **(A)** The hierarchical clustering tree is based on the *in vitro* protein expression values. The control, epirubicin, doxorubicin, and idarubicin samples were colored by red, blue, green, and purple, respectively. **(B)** The module eigengene (ME) values in WGCNA modules. **(C)** The PCA of the modules turquoise, blue, and green. Drugs: DOX, doxorubicin; EPI, epirubicin; IDA, idarubicin. Treatment doses: The, therapeutic dose; Tox, toxic dose. Exposure period: 002: 2 h exposure, 008: 8 h exposure, 024: 24 h exposure, 072: 72 h exposure, 168: 168 h exposure, 240: 240 h exposure, 336: 336 h exposure.

In total, 2,497 proteins were detected among 139 cardiac microtissue samples (FDR = 5%) with 2,327 proteins having a unique UniProt ID. In these unique UniProt ID proteins, after removing proteins with over 50% missing values across samples, the remaining 810 proteins were used to construct the hierarchical clustering samples tree (Figure 3A). The control samples grouped in a separate branch, while samples treated by ANTs clustered in another branch. Among the ANTs samples, IDA-treated samples converged at one sub-branch, whilst samples treated with DOX and EPI are in two closely connected sub-branches, except for the samples treated with the EPI toxic dose at late time points (168, 240, and 336 h exposure).

The WGCNA divided these 810 proteins by their expression profiles over drugs, doses, and time into 6 modules named by colors. The large modules were the turquoise and blue modules, which consisted of 262 and 194 proteins, respectively, while other modules (brown, yellow, green, and red) consisted of smaller numbers of proteins (148, 96, 80, and 30 proteins, respectively) (Table 1). In the turquoise and blue modules, their eigengene values (the first principle component) demonstrated distinctions in protein expression levels between ANT-treated samples and control samples, while this did not happen in other modules (Figure 3B and Supplementary Figure 1). However, the PCA plots indicated that protein profiles in the green module also showed a separation between ANT-treated samples and control samples (Figure 3C and Supplementary Figure 2). By using module eigengene values and PCA, we identified 3 protein modules (turquoise, green, and blue) in which the difference in protein expressions between ANTs and control conditions was evident. The high module membership proteins in the *in vitro* protein modules were identified as weighted proteins (Table 1).

**TABLE 1:** The number of proteins in WGCNA modules in the *in vitro* dataset.

	Total proteins	Turquoise module	Blue module	Brown module	Yellow module	Green module	Red module
Number of proteins	810	262	194	148	96	80	30
Number of proteins related to heart failure	101	25	14	12	4	7	0
Number of the high module membership proteins	–	10	8	19	3	3	0
Number of the high module membership proteins related to heart failure	–	1	0	3	0	0	0

Subsequently, the pathway analysis in ConsensusPathDB (*p*-value < 0.01, *q*-value < 0.01) indicated that the proteins in the turquoise and blue modules are mainly involved in translation and protein metabolism pathways. The pathway analysis performed on only weighted proteins in the turquoise and blue modules showed a similar outcome (Supplementary Table 3). While the proteins in the green module are related to the metabolism pathways, the number of weighted proteins in this module was small (3 proteins) and appeared non-overrepresented in any pathway (Supplementary Table 3). Multiple proteins belonging to these modules are known as heart failure-related proteins in the DisGeNET database (Table 1).

### Human Biopsies Data

**FIGURE 4:**
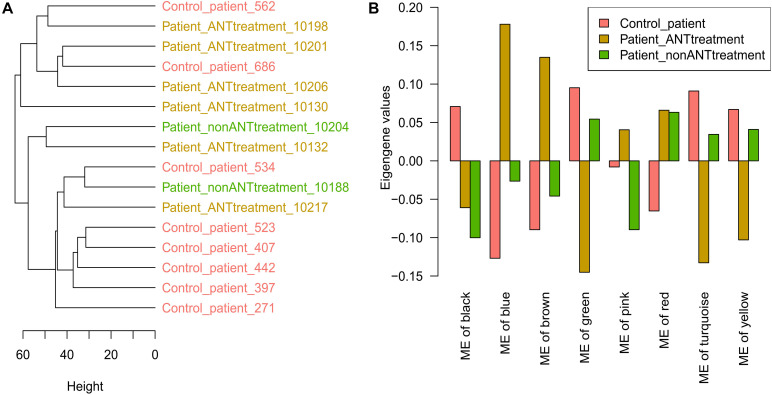
Human cardiac biopsies proteomics data analysis. **(A)** The hierarchical clustering tree is based on the protein expression values in biopsy samples. **(B)** The module eigengene (ME) values in WGCNA modules. Control_patient: heart failure patients with no cancer history; Patient_ANTtreatment: heart failure patients that had cancer treatment with anthracyclines (ANTs); Patient_nonANTtreatment: heart failure patients that had cancer treatment without ANTs; the number at the end of each patient indicates the biopsies sample ID.

In 21 cardiac biopsies from heart failure patients, there were 1,639 proteins with a unique UniProt ID out of 1,669 proteins that were detected (FDR = 5%). Five biopsies from 2 patients that had no cancers and 3 patients that had cancer and received ANT treatments were used to establish the biopsies preparation workflow before the biopsies preparation workflow was applied to the remaining 16 biopsy samples. Therefore, the first 5 biopsies showed large distances to the latter 16 biopsy samples in the hierarchical clustering sample tree (Supplementary Figure 3). The latter 16 biopsy samples, including 8 patients that had no cancers as a control group, 6 patients that had undergone cancer treatment including ANTs, and 2 patients that had received cancer treatment without ANTs, were used for further analysis. Proteins with over 50% missing expression values across samples were removed, and the remaining 1,602 proteins were used for further analysis. The hierarchical clustering sample tree built from the 16 samples could not define patient groups according to their medical history (Figure 4A). The PCA plot also confirmed that there was no apparent difference between cardiac biopsy samples taken from the different patient groups (Supplementary Figure 4).

The WGCNA analysis divided the 1,602 proteins into 8 modules based on their expression pattern across the patient groups. Although these modules consisted of different protein sets, 6 of them were also named by the same colors as the *in vitro* data analysis. The large modules were the turquoise, blue, and brown modules, which consisted of 342, 330, and 320 proteins, respectively, while other modules (yellow, green, red, black, and pink) consisted of smaller numbers of proteins (Table 2). In the turquoise, blue, brown, yellow, and green modules, their eigengene values manifested the difference in protein expressions between control patients and patients who had undergone cancer treatment including ANTs treatment (Figure 4B). The PCA plot did not identify additional modules that showed differences in protein expression between ANT-treated and control groups (Supplementary Figure 4). Weighted proteins were defined from the high module membership proteins in each module (Table 2).

**TABLE 2:** The number of proteins in WGCNA modules in the human biopsies dataset.

	Total proteins	Turquoise module	Blue module	Brown module	Yellow module	Green module	Red module	Black module	Pink module
Number of proteins	1,602	342	330	320	213	177	135	47	38
Number of proteins related to heart failure	159	22	22	21	18	9	10	7	2
Number of the high module membership proteins	–	115	95	65	54	53	41	13	5
Number of the high module membership proteins related to heart failure	–	11	8	8	4	3	5	1	1

The pathway analysis indicated that the proteins in these 5 selected modules from the biopsies data are related to different cellular mechanisms comprising metabolism, mitochondrial function, muscle contraction, and signaling pathways. The pathway analysis for only weighted proteins in these modules showed a similar outcome (Supplementary Table 4). According to the DisGeNET database, multiple proteins in these selected modules are known as heart failure-related proteins (Table 2).

### Combining the *in vitro* and Human Biopsies Data

Combining proteins used in the *in vitro* cardiac microtissue and human biopsies analysis (810 and 1,602 proteins, respectively) resulted in 704 proteins present in both datasets. Of these, 242 proteins (34.4%) were weighed in WGCNA modules in at least one dataset, and 7 of them were weighted proteins in both datasets (Table 3). From the 7 weighted proteins in both datasets, DECR1, SH3BGRL, and ATP5F1B have been recognized as heart failure-related proteins (Narain et al., 2014; Piñero et al., 2019; Ali et al., 2020). Although the role of ETFB in the late stages of heart failure is not clear yet, a cohort study showed that ETFB is strongly associated with chronic ANT-induced cardiotoxicity (Ruiz-Pinto et al., 2018). The remaining proteins, i.e., EEF1D, TIMM13, and PMPCB, are involved in transferring aminoacyl-tRNAs, regulating heat-shock response, and maintaining mitochondrial functions (The UniProt Consortium, 2018), but their roles in heart disease contexts have not been investigated.

**TABLE 3:** The high module membership proteins in both *in vitro* and human biopsies datasets.

		ANTs—related modules in the *in vitro* data		
		
		Turquoise	Brown	Green
ANTs—related modules in the biopsies data	Turquoise	–	–	–
	Blue	SH3BGRL (O75368) EEF1D (P29692)	–	–
	Brown	TIMM13 (Q9Y5L4)	ATP5F1B (P06576) ETFB (P38117) DECR1 (Q16698)	PMPCB (O75439)
	Yellow	–	–	–
	Green	–	–	–

**The protein names were retrieved from UniProt ID.**

**FIGURE 5:**
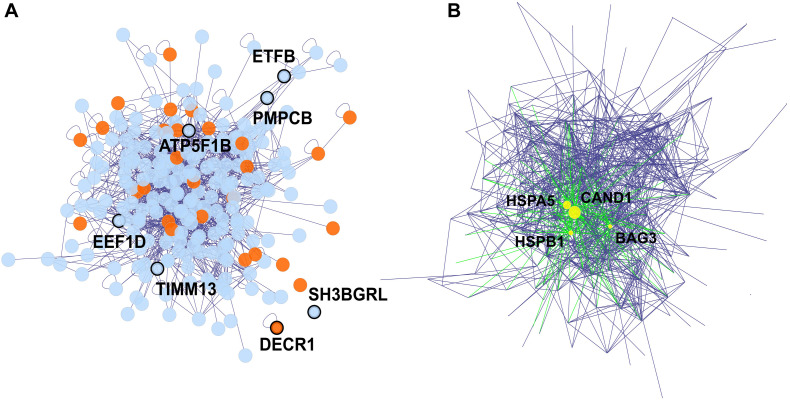
The protein-protein interaction network of proteins detected in both *in vitro* and biopsies datasets, and that were weighted proteins in at least one dataset. Edges represent the protein-protein interaction, and nodes represent proteins, 16 orphan nodes were removed from the network. **(A)** The 7 nodes with protein names in bold and showing thicker border were weighted protein in both *in vitro* and biopsies dataset. Proteins associated with heart failure (detected via DisGeNET), were highlighted in orange. **(B)** The 4 hub proteins (>30 degrees of connection) of the protein-protein interaction network are shown as yellow nodes.

The 242 proteins which were weighted proteins in at least one dataset, were used to establish a protein-protein interaction network via the BisoGenet app in Cytoscape (Figure 5). In this network, 28 proteins (11.57%) are known as heart failure-related proteins (Figure 5A). We identified 4 hub proteins which are nodes with a high degree of connectivity and are key connectors between proteins in the network: CAND1, HSPA5, HSPB1, and BAG3 (Figure 5B and Supplementary Figure 5). Literally, these hub proteins are essential components in the proteome: CAND1 contributed to the ubiquitin complexes, HSPA5 and HSPB1 are heat shock proteins involved in stress responses, while BAG3 is a co-chaperone for HSP70 and HSC70 chaperone proteins (The UniProt Consortium, 2018). The alterations in these hub protein expressions under ANT treatment can systematically spread across the protein network, and lead to the cascade effect of ANTs.

### Expression of the Weighted Proteins and the Hub Proteins

**FIGURE 6:**
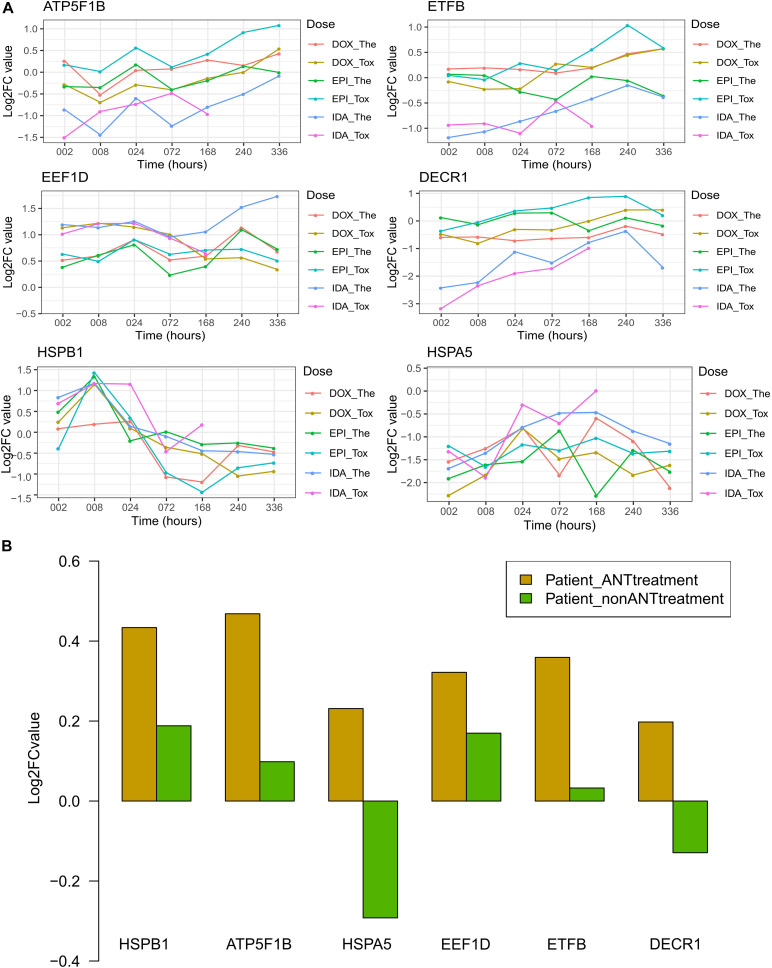
The log2FC expression of selected proteins in the *in vitro* and human biopsies datasets. Protein names and its UniPort IDs: ATP5F1B (P06576), EEF1D (P29692), ETFB (P38117), DECR1 (Q16698), HSPA5 (P11021), and HSPB1 (P04792). **(A)** The log2FC value of *in vitro* data resulted from the comparison of protein expression levels in anthracyclines (ANTs) samples to control samples per time point. **(B)** The log2FC value of human biopsies data resulted from the protein expression levels in heart failure patients having cancer therapy with or without ANTs compared to the heart failure patients with no cancer (control group). Patient_ANTtreatment: heart failure patients that had cancer treatment with ANTs; Patient_nonANTtreatment: heart failure patients that had cancer treatment without ANTs.

Of these 7 weighted proteins in both datasets and 4 hub proteins, their expression level varied over time across ANT treatment conditions in the *in vitro* data, as well as varied across patient groups in the biopsies data. Of these 11 proteins, 6 proteins i.e., ATP5F1B, EEF1D, ETFB, DECR1, HSPA5, and HSPB1, had high expression (log2 expression > 10) in all samples (Supplementary Figure 6). The log2 fold change (log2FC) is the log-ratio of protein expression in ANT treatments compared to the protein expression in equivalent control samples. In the *in vitro* testing, log2FCs of these 6 proteins were used to manifest the up- and down-regulated proteins overtime under ANT-effects after correcting for the correspondent baseline in control samples (Figure 6A). Regarding the human heart biopsy samples, the log2FC of these 6 proteins represents differences between cancer patients who received ANT and non-ANT treatment vs. non-cancer patients (Figure 6B).

Generally, the *in vitro* protein expression in IDA-treated samples differed from DOX- and EPI-treated samples, especially for the 4 proteins i.e., ATP5F1B, ETFB, EEF1D, and DECR1 (Figure 6A). The DECR1 and ETFB expression in the IDA-treated samples was downregulated at early time points, then increased and were closer to their expression in DOX and EPI-treated samples (Figure 6A). These 2 proteins show that the effect of ANT analogs could be diverse in short-term treatments, but become more convergent after long-term treatment. The log2FCs of ATP5F1B in ANTs-treated samples increased at later time points (from 168 to 336 h), despite their fluctuations at earlier time points in the *in vitro* data (Figure 6A). Interestingly, this is in agreement with the higher expression levels of ATP5F1B in the heart biopsies taken from patients treated with ANTs compared to control patients and patients treated without ANTs (Figure 6B). In addition, although the log2FCs of EEF1D differed between *in vitro* ANTs-treated samples, their log2FCs were always positive (Figure 6A). Similarly, EEF1D was overexpressed in the patients treated with ANTs compared to control patients and patients treated without ANTs (Figure 6B). For the other 4 proteins (ETFB, DECR1, HSPB1, and HSPA5), the *in vitro* data did not clearly show the same pattern as apparent in the human biopsies data (Figure 6).

## Discussion

Recent researches on ANTs have explored the ANT-effects on cellular molecules such as DNA (Silva et al., 2017) and RNA (Reyes et al., 2017), but little is known about the ANT effects on the proteome level. In this study, we investigated how ANTs alter the protein expressions, influence cellular mechanisms, and promote heart failure in human cardiac microtissue as well as in human cardiac biopsies from ANTs-treated patients.

The *in vitro* clustering sample tree was built based on protein expressions in cardiac microtissues (Figure 3A). In the clustering tree, samples were grouped according to their exposure profiles. The *in vitro* DOX and EPI-treated samples were particularly showing a high similarity in protein expressions not only in the clustering tree but also in module eigengene values and PCA plots (Figures 3A–C). This confirms previous claims that EPI is a derivative of DOX, and shares a similar mechanism with DOX, while IDA is an analog derived from daunorubicin, another ANT (Kuffel et al., 1992; Minotti et al., 2004). The lipophilic ability could be an underlying factor that leads to the differences in protein profiles, in which IDA has higher lipophilicity than its parent molecule, daunorubicin (Matyszewska, 2020), while daunorubicin has higher lipophilicity than DOX (Matyjaszczyk-Gwarda et al., 2019). Specially, a rat cardiomyoblast study showed the inverse correlation between the lipophilicity of ANT analogs and their toxicity (Matyjaszczyk-Gwarda et al., 2019).

While the *in vitro* model, as a well-controlled system, seems to be able to capture subtle divergences across ANT analogs, the human biopsies could not be grouped by the protein profiles in accordance with the patients’ medical history (Figure 4A). The main reason could be that the participants were all heart failure patients, who share quite similar protein expression profiles. Furthermore, clinical treatments usually incorporate different ANT analogs with other drugs such as docetaxel, cytosine arabinoside, vincristine, etc., rather than using a single ANT as a monotherapy (Supplementary Table 2). This combined chemotherapy has been recommended because of its survival benefit and cost-effectiveness (Keating et al., 1981; von Minckwitz, 2007). However, it also causes challenges for ANT-induced cardiotoxicity study. Even though patient data is certainly complex and reflects multifactor treatments, the combination of *in vitro* and human biopsies data could still be used to project the outcome of an empirical experiment to clinical applications. Our study design thus represents an innovative approach and may therefore contribute toward the relevance of dedicated *in vitro* models for predicting human health outcomes.

Proteins in the *in vitro* and human biopsies data were grouped into different modules according to their expression. In the *in vitro* data, 3 protein groups (turquoise, green, and blue) represented a part of the proteome induced by ANTs (Figures 3B,C). These groups demonstrated not only how certain ANT analogs but also how different doses and time exposure can impact protein expressions. In the PCA plots, while samples from short-time exposure were grouped, samples treated with toxic doses and from longer exposures (168, 240, and 336 h) were more dispersed (Figure 3C). An early clinical study showed that ANTs possibly cause myocardial damage after 24 h following drug administration; however, patients may recover after 72 or 96 h (Singer et al., 1978). Related to this clinical phenomenon, the proteome observed in the *in vitro* ANTs samples at early time points could reflect the acute ANTs toxicity, while the proteome observed at later time points represented the intermediate and late toxicity. Thus, evaluating the proteome after long-term exposure such as 168 h (1 week) might elucidate more relevant aspects of ANT chronic cardiotoxicity. This approach also has been proposed in another study that predicts ANT cardiotoxicity in patients before the obvious clinical symptoms develop using a serial assessment of the left ventricular function in 1–3 weeks after treatment (Singer et al., 1978; Ritchie et al., 1980). In the biopsies, 5 protein groups (turquoise, blue, brown, yellow, and green modules) highlighted the difference in the protein expressions between the heart failure patients groups (Figure 4B).

Protein groups, that represented ANT-induced alteration of protein expressions, were functionally annotated to particular biological processes. From the classical point of view, ANTs, as reactive oxygen species generators, can affect the cardiac iron-dependent and iron-independent mechanisms, then disturb the metabolism pathway (Menna et al., 2007; Gammella et al., 2014). In this study, a part of the ANTs-affected proteins was involved in the metabolism pathway in both the *in vitro* and cardiac biopsy samples (Supplementary Tables 3, 4). It seems that ANT treatment causes not only acute effects but also chronic effects on cellular metabolisms. Additionally, the ANTs-affected proteins in the *in vitro* dataset also belonged to the translation pathway (Supplementary Table 3). Research has indicated that DOX alters the transcription process via signaling factors such as the transcription factor NF-κB, the transcription factor GATA4, or through the PI3K-dependent signaling pathway (Ghigo et al., 2016). Furthermore, the ANTs-affected proteins in the human biopsies dataset illustrated the impact of the ANT treatment on the mitochondrial function, muscle contraction, and signaling pathways in the long term (Supplementary Table 4). While mitochondrial dysfunction has been a longitudinal topic in investigating ANT-side effects (Murabito et al., 2020), signaling pathways have especially emerged as a new paradigm of ANT-induced cardiotoxicity (Ghigo et al., 2016).

The 704 proteins were detected in both the *in vitro* and the biopsies datasets, and 7 of them were weighted protein in both datasets (Figure 5 and Table 3). These weighted proteins suggest an extrapolation from *in vitro* outcomes to *in vivo*, in which they demonstrated strong impacts both in the *in vitro* samples as well as in human biopsies. One of these weighted proteins was ATP5F1B, which is an ATP synthase subunit beta protein and belongs to the ATP synthase complex (The UniProt Consortium, 2018). ATP5F1B was up-regulated in both *in vitro* and biopsy samples (Figure 6) suggesting compensation of the mitochondrial disfunction under ANT treatment. However, the ATP levels still decreased in all *in vitro* ANTs samples after long-term exposure (Figures 1E–G). EEF1D, another weighted protein, is involved in transferring aminoacyl-tRNAs to the ribosome and regulating heat-shock-responsive genes (The UniProt Consortium, 2018). This protein was triggered by ANTs and up-regulated from the beginning of the *in vitro* ANT treatments as well as in the ANTs-treated heart failure patients (Figure 6A). The consistency in ATP5F1B and EEF1D’s expression pattern between the *in vitro* samples and human cardiac biopsies suggests that these two proteins could be potential targets to predict ANT-induced cardiotoxicity.

The other 5 weighted proteins also play important roles in cellular function and are associated with heart failure. DECR1, an enzyme in the mitochondrial fatty acid beta-oxidation pathway (2,4-dienoyl-CoA reductase), has been known as a heart failure-related protein in the DisGeNET database (Piñero et al., 2019). The promoter of weighted protein SH3BGRL contains an NF-κB binding site bounded, thus it can be regulated by the Rel/NF-κB family (Majid et al., 2006); while the activation of NF-κB can be modulated by ANTs (Ghigo et al., 2016). Furthermore, a clinical study showed that SH3BGRL was differentially expressed on the transcriptomic level between heart failure biopsies of non-ischemic cardiomyopathy patients and non-heart failure biopsies from unused cardiac transplant donors (Kittleson et al., 2005). Even though it is unclear how SH3BGRL is directly involved in heart failure, SH3BGRL has evolved into a biomarker for identifying cardiotoxic agents and for diagnosing heart diseases (Narain et al., 2014). The other weighted proteins, i.e., TFB, PMPCB, and TIMM13, are mitochondrial proteins (Figure 6), are mediated by TLR4 and NF-κB activation, which can be triggered by ANT (Schilling et al., 2011; Ghigo et al., 2016). ETFB is involved in mitochondrial electron transfer and is strongly associated with chronic anthracycline-induced cardiotoxicity (Ruiz-Pinto et al., 2018). PMPCB (aka β-MPP) belongs to the mitochondria proteases, while TIMM13 is a chaperone-like protein; both of them are essential for importing and modulating proteins in mitochondria (Bota and Davies, 2001; Gottlieb and Thomas, 2017; The UniProt Consortium, 2018).

The protein-protein network represents inter-dependence interactions between heart failure-related protein and other proteins (Figure 5). Four hub proteins in the network were CAND1, HSPA5, HSPB1, and BAG3 (Figure 5B), which serve as key bridges for protein-protein interactions. CAND1 is a key assembly factor of SCF E3 ubiquitin ligase complexes (Pierce et al., 2013), which manipulate the turnover and function of the sarcomere proteins and the apoptosis signaling pathway in cardiomyocytes. The alterations in ubiquitin-proteasome (E3s) are associated with cardiac dysfunction (Willis et al., 2014). Another hub protein, HSPA5, is a member of the heat shock protein family A (Hsp70). This protein maintains protein homeostasis and Ca^2+^ homeostasis in the endoplasmic reticulum, as well as activates the unfolded protein response pathway and induces autophagy. HSPA5 is a current target for protecting cardiomyocytes because it is an oxidative stress sensor and responder and can rescue cardiomyocytes from apoptosis (Wang et al., 2017). The HSPA5 level under ANT treatment was fluctuated since early time exposures (Figure 6), while HSPA5 was expected to be gradually changed due to its long half-life (over 30 days) (Wang et al., 2017). This suggests that HSPA5 expression was immediately impacted by ANTs, and could not protect cardiomyocytes, which lead to chronic injury of heart tissue. Another heat shock protein, i.e., HSPB1, participates in different cell functions including stress resistance (The UniProt Consortium, 2018). HSPB1 was up-regulated in mice cardiomyocytes as a response to myocardial infarction and involved in repairing tissue damaged by inhibiting NFκB inflammatory signaling (Wang et al., 2018). In this study, HSPB1 levels were up-regulated in all *in vitro* ANT-treated samples in early time exposure (2–24 h) (Figure 6A), which may indicate an acute response to ANT treatment as a stress responder. However, after 24 h, the HSPB1 expression decreased in all ANT-treated samples (Figure 6A). The last hub protein is BAG3, which is a co-chaperone for HSP70 and HSC70 chaperone proteins and is involved in a wide range of different cell functions (The UniProt Consortium, 2018). BAG3 has been reported to maintain cardiomyocyte function during proteotoxic stress and may become a target for heart failure therapy in the clinic (Feldman et al., 2017; Judge et al., 2017).

In conclusion, this study provided a broad picture of protein profiles under ANTs exposure. The *in vitro* cardiac microtissues experiment showed a distinction of protein profiles between early and later exposure, which suggests different cellular responses for acute and intermediate/chronic ANT toxicity. A part of the proteins shows similar expression patterns between the *in vitro* cardiac microtissues and human biopsies under ANT exposure. Some of these proteins belong to traditional ANT-affected pathways, while other proteins are involved in signaling pathways, which have emerged in recent ANT toxic studies. This study demonstrated 7 weighted proteins detected in both datasets and 4 hub proteins detected from the protein-protein network. The function of these 11 proteins indicates the effects of ANTs in the mitochondria functions and signaling pathways, especially the NF-κB signaling pathway which is a prominent factor in the recent ANT-induced cardiotoxicity paradigms. Of these 11 proteins, SH3BGRL, HSPA5, and BAG3 are current cardiotoxicity biomarkers or targets of cardiac therapy. Other proteins, such as ATP5F1B and EEF1D, were consistently up-regulated in the *in vitro* ANTs-treated samples after 1 week of exposure as well as in biopsies taken from patients treated with ANTs. While ATP5F1B reflects how ANTs could promote mitochondrial dysfunction, EEF1D is a stress responder and can trigger other heat-shock genes. These proteins could be potential biomarkers for cardiotoxicity and may reveal new insights into the proteome alterations caused by ANTs in the *in vitro* human cardiac tissues and translate to the patient situation.

## Data Availability Statement

The data presented in the study are deposited in the BioStudies repository (http://www.ebi.ac.uk/biostudies) accession number S-HECA104 for *in vitro* proteomics data and S-HECA50 for human biopsies proteomics data. Further inquiries can be directed to the corresponding author.x1

## Ethics Statement

The studies involving human participants were reviewed and approved by the Medical Ethics Committee of Maastricht University Medical Center. The patients/participants provided their written informed consent to participate in this study.

## Author Contributions

NN performed the proteomic data analysis and drafted the manuscript. TS conceived of the proteomic data analysis and helped to draft the manuscript. MV previously worked with related (transcriptomics) datasets generated from the same microtissues and was a consultant regarding the study design. HG performed the peptide/protein quantification and preprocessing data. NS and RS carried out the sample preparation and mass spectrometry measurements. JK supervised the study and contributed to writing and correcting the manuscript. DJ supervised the study and contributed to data interpretation and to writing and correcting the manuscript. All authors read and approved the final manuscript.

## Conflict of Interest

The authors declare that the research was conducted in the absence of any commercial or financial relationships that could be construed as a potential conflict of interest.

## Supplementary Material

Supplementary Figure 1 Module eigengene (ME) values in all *in vitro* modules.

Supplementary Figure 2 PCA plots in all *in vitro* modules.

Supplementary Figure 3 Hierarchical clustering tree with all samples in the human cardiac biopsies dataset. The samples in the red rectangular belonged to one batch, while the rest of the samples belonging to another batch, were considered as outliers and were removed for further analysis. Control_patient: heart failure patients with no cancer history; Patient_ANTtreatment: heart failure patients that had cancer treatment with anthracyclines (ANTs); Patient_nonANTtreatment: heart failure patients that had cancer treatment without ANTs; the number at the end of each patient indicates the biopsies sample ID.

Supplementary Figure 4 PCA plot from all proteins in the biopsies dataset and from proteins in each biopsies module. Patient_ANTtreatment: heart failure patients that had cancer treatment with anthracyclines (ANTs); Patient_nonANTtreatment: heart failure patients that had cancer treatment without ANTs. The number of cancer patients treated without ANT was small, so there was no ellipse calculation for them.

Supplementary Figure 5 Protein network analysis: the number of nodes with their degree of connection. Four hub proteins (degree of connection ≥ 30) were highlighted in black: CAND1 (Q86VP6)—57 connections, HSPA5 (P11021)—43 connections, HSPB1 (P04792)—32 connections, and BAG3 (O95817)—30 connections.

Supplementary Figure 6 The log expression of selected proteins in **(A)** the *in vitro* and **(B)** human biopsies datasets. Protein names and its UniPort IDs: ATP5F1B (P06576), EEF1D (P29692), ETFB (P38117), DECR1 (Q16698), HSPA5 (P11021), and HSPB1 (P04792).

Supplementary Table 1 The concentrations of anthracycline in the *in vitro* samples.

Supplementary Table 2 The demographic and clinical information of the biopsies dataset.

Supplementary Table 3 The top 10 over-represented pathway of selected modules in the *in vitro* data (*p*-value < 0.01, and *q*-value < 0.01).

Supplementary Table 4 The top 10 over-represented pathway of selected modules in the human biopsies data (*p*-value < 0.01, and *q*-value < 0.01).
