# Supplementary material for: Translational Proteomics Analysis of Anthracycline-Induced Cardiotoxicity From Cardiac Microtissues to Human Heart Biopsies
Source: Front Genet. 2021 Jun 15;12:695625. doi: 10.3389/fgene.2021.695625 (PMC8239409; doi:10.3389/fgene.2021.695625)
Supplement: Supplementary Figure 1 — Module eigengene (ME) values in all in vitro modules. [file Presentation_1.PDF]

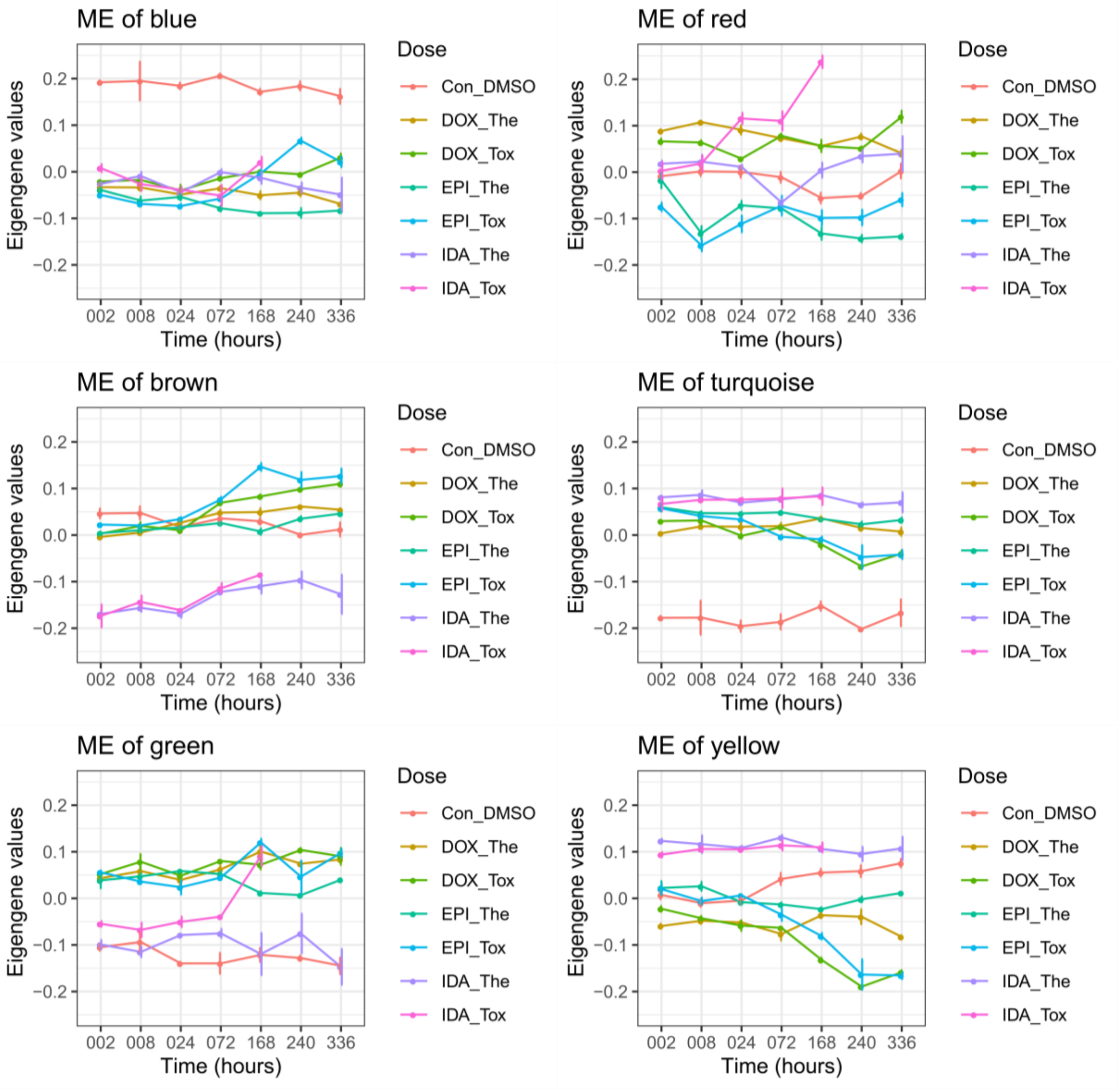

Figure S1: Module eigengene (ME) values in all *in vitro* modules

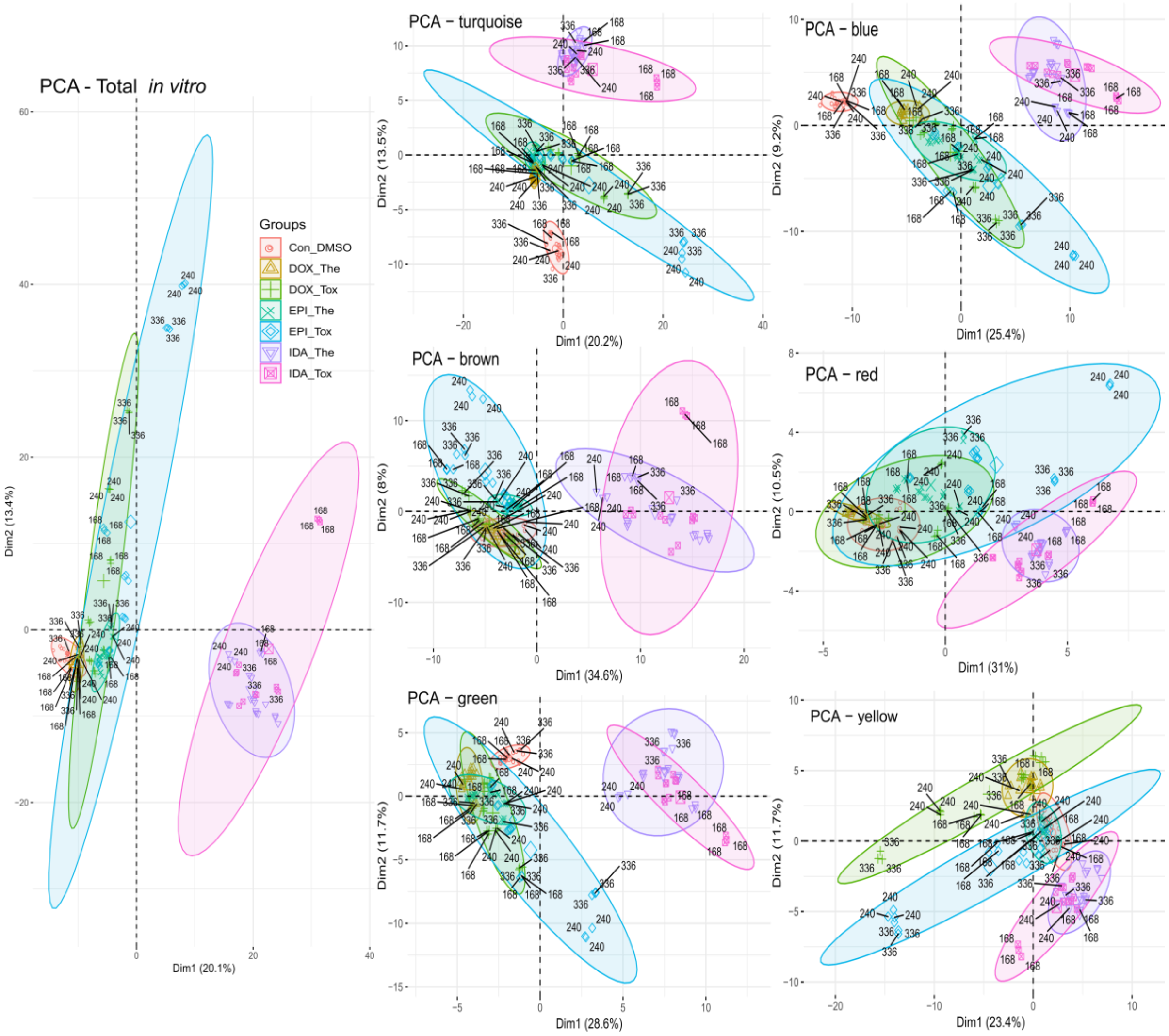

Figure S2: PCA plots in all *in vitro* modules

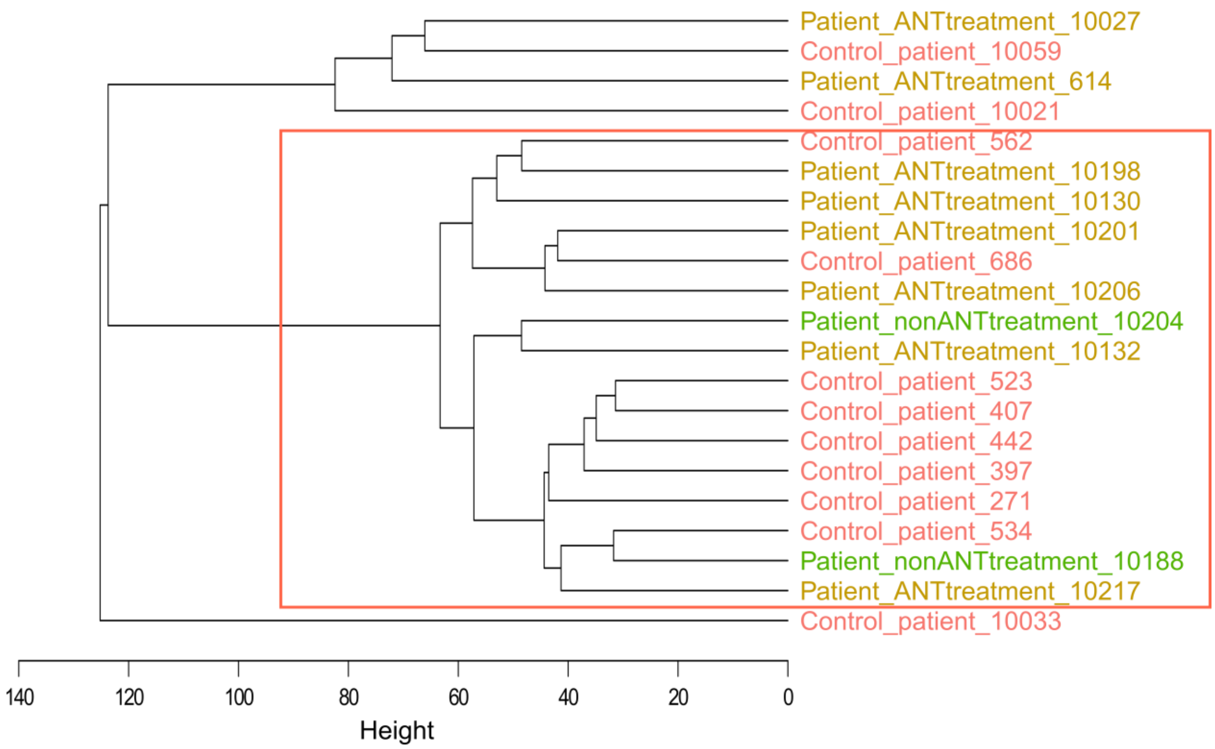

Figure S3: Hierarchical clustering tree with all samples in the human cardiac biopsies dataset. The samples in the red rectangular belonged to one batch, while the rest of the samples belonging to another batch, were considered as outliers and were removed for further analyses. Control_patient: heart failure patients with no cancer history; Patient_ANTtreatment: heart failure patients that had cancer treatment with anthracyclines (ANTs); Patient_nonANTtreatment: heart failure patients that had cancer treatment without ANTs; the number at the end of each patient indicates the biopsies sample ID.

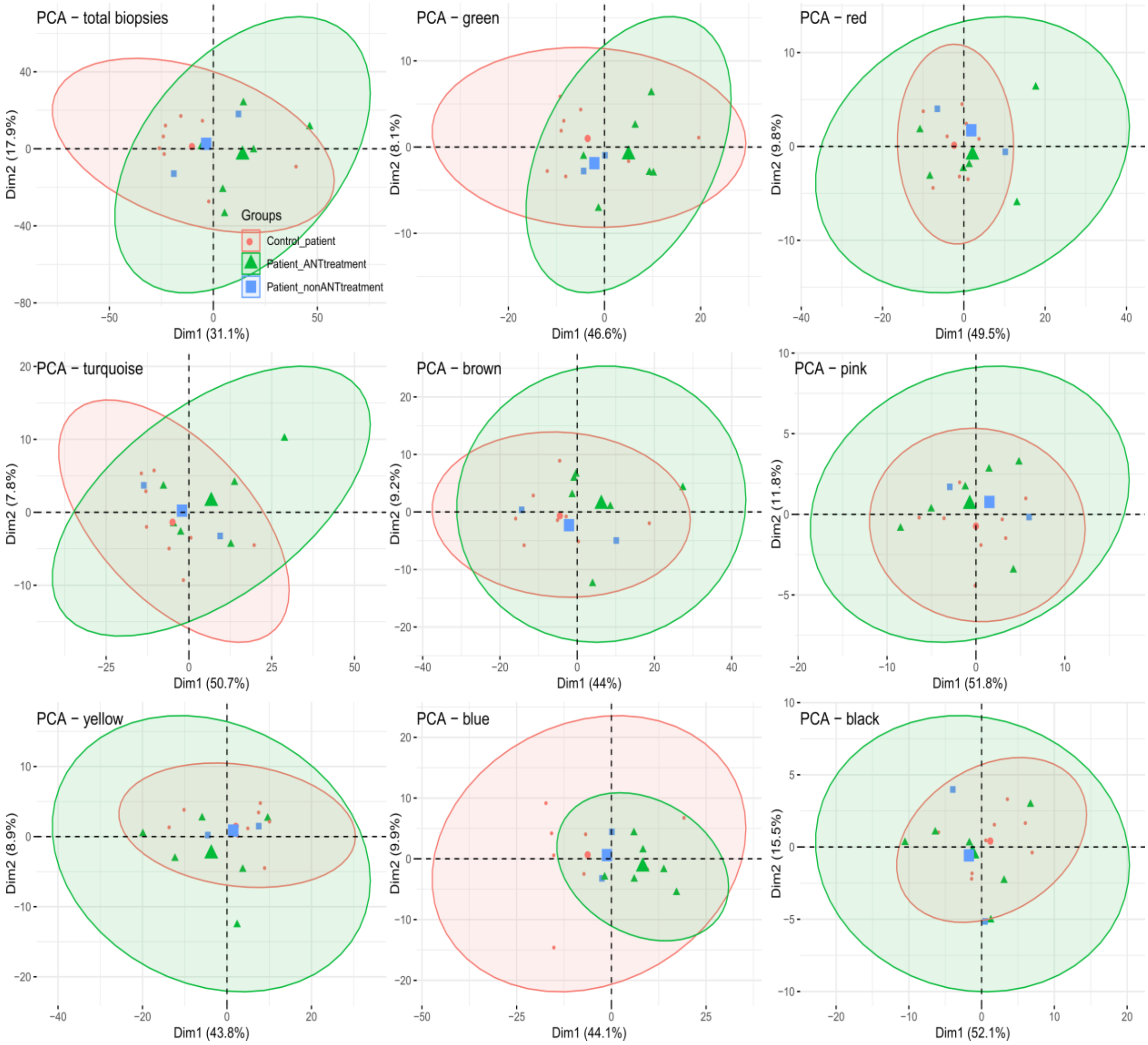

Figure S4: PCA plot from all proteins in the biopsies dataset and from proteins in each biopsies module. Patient_ANTtreatment: heart failure patients that had cancer treatment with anthracyclines (ANTs); Patient_nonANTtreatment: heart failure patients that had cancer treatment without ANTs. The number of cancer patients treated without ANT was small, so there was no ellipse calculation for them.

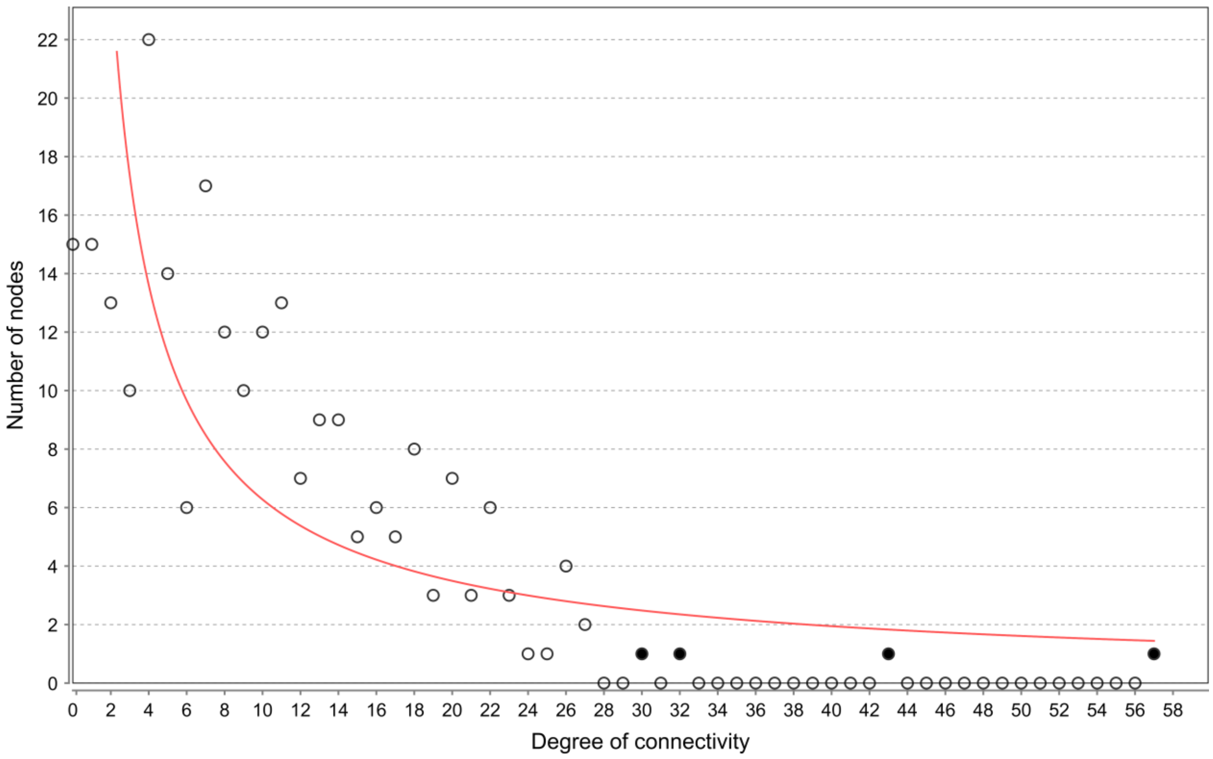

Figure S5: Protein network analysis: the number of nodes with their degree of connection. Four hub proteins (degree of connection >= 30) were highlighted in black: CAND1 (Q86VP6) – 57 connections, HSPA5 (P11021) – 43 connections, HSPB1 (P04792) – 32 connections, BAG3 (O95817) – 30 connections.

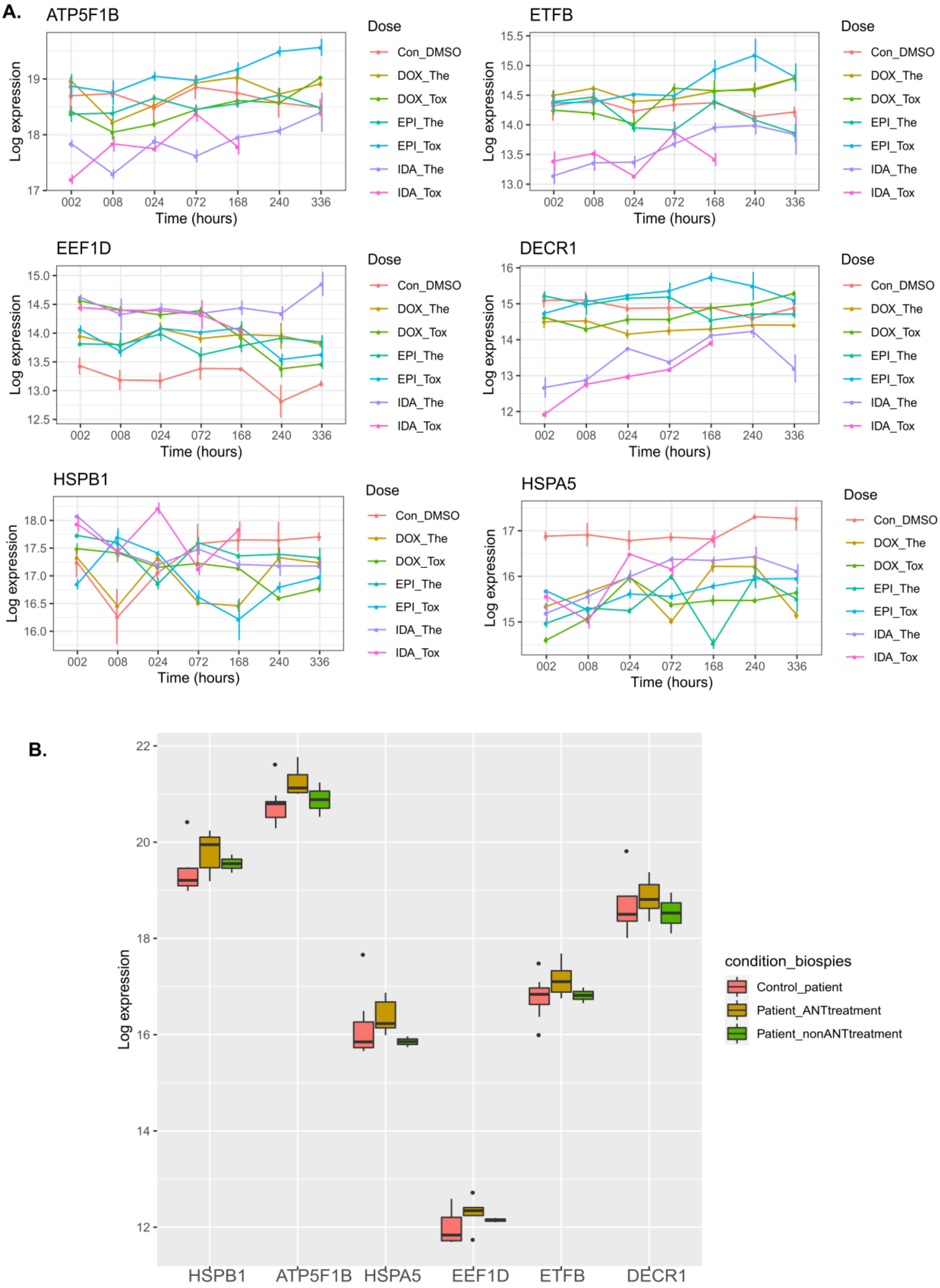

Figure S6: The log expression of selected proteins in the *in vitro* and human biopsies datasets. Protein names and its UniPort IDs: ATP5F1B (P06576), EEF1D (P29692), ETFB (P38117), DECR1 (Q16698), HSPA5 (P11021), HSPB1 (P04792).
